# An Enzymatically Hydrolyzed Animal Protein-Based Biostimulant (Pepton) Increases Salicylic Acid and Promotes Growth of Tomato Roots Under Temperature and Nutrient Stress

**DOI:** 10.3389/fpls.2020.00953

**Published:** 2020-07-01

**Authors:** Andrea Casadesús, Marina Pérez-Llorca, Sergi Munné-Bosch, Javier Polo

**Affiliations:** ^1^ Department of Evolutionary Biology, Ecology and Environmental Sciences, University of Barcelona, Barcelona, Spain; ^2^ Research Institute of Nutrition and Food Safety (INSA), University of Barcelona, Barcelona, Spain; ^3^ R&D Department, APC Europe S.L., Granollers, Spain

**Keywords:** auxin, biostimulants, chorismate-derived hormones, melatonin, PIN proteins, salicylic acid, tomato plants

## Abstract

Biostimulants may be particularly interesting for application in agricultural and horticultural crops since they can exert a growth-promoting effect on roots. This may be important for promoting longitudinal and lateral root growth and therefore increasing belowground vegetative growth, which may in turn lead to improved aboveground vegetative growth and increased yields. Here, we examined the effects and mechanism of action of an enzymatically hydrolyzed animal protein-based biostimulant (Pepton) on the root growth of tomato plants, with an emphasis on its possible role on chorismate-derived hormones (auxin, salicylic acid, and melatonin). Tomato plants growing in hydroponic systems were exposed to either nutrient stress conditions (experiment 1) or suboptimal temperatures (experiment 2) in a greenhouse, and the concentration of auxin, salicylic acid, and melatonin in roots were measured just prior and after the application of the biostimulant. Results showed that the application of Pepton exerted a growth-promoting effect on roots in plants growing under suboptimal conditions, which might be associated with enhanced salicylic acid levels in roots. The extent of effects of this enzymatically hydrolyzed animal protein-based biostimulant might strongly depend on the growth conditions and stage of root system development. It is concluded that an enzymatically hydrolyzed animal protein-based biostimulant (Pepton) may exert a positive effect enhancing primary and lateral root growth of tomato plants growing under suboptimal conditions, by stimulating the biosynthesis of specific hormonal pathways, such as salicylic acid under stress.

## Introduction

Recently, much effort is being put worldwide to boost research focused on the environmentally friendly biostimulation of crop performance for improving plant production in the frame of sustainable farming management. Protein hydrolysates, and most particularly those obtained from recycling wastes of plant or animal origin, are good candidates to be used as plant biostimulants because of their high amino acids and soluble peptide concentrations (Sestili et al., 2018; Caruso et al., 2019). Among several protein hydrolysates used as biostimulants, enzymatically hydrolyzed animal protein-based biostimulants such as Pepton 85/16® (Pepton), represent a cost-effective approach to alleviate the negative effects of several stresses in horticultural crops (Polo et al., 2006; Polo and Mata, 2018; Casadesús et al., 2019). Furthermore, the application of Pepton at different levels (2 to 4 kg/ha) has been shown to improve linearly root length and several vegetative growth parameters thus increasing yield by 27% compared with the control treatment in cherry tomatoes under low stress ambient field conditions (Polo and Mata, 2018). However, still little is known about the possible effects exerted by this biostimulant on root growth and even less about its mechanism of action, although it seems that the positive effects of Pepton on horticultural crops may be exerted by the presence of amino acids, which can influence the physiology of plants both directly or indirectly, the latter through the stimulation of various metabolic processes, such as an increased production of growth-promoting hormones in aboveground vegetative tissues (Casadesús et al., 2019).

**Figure 1:**
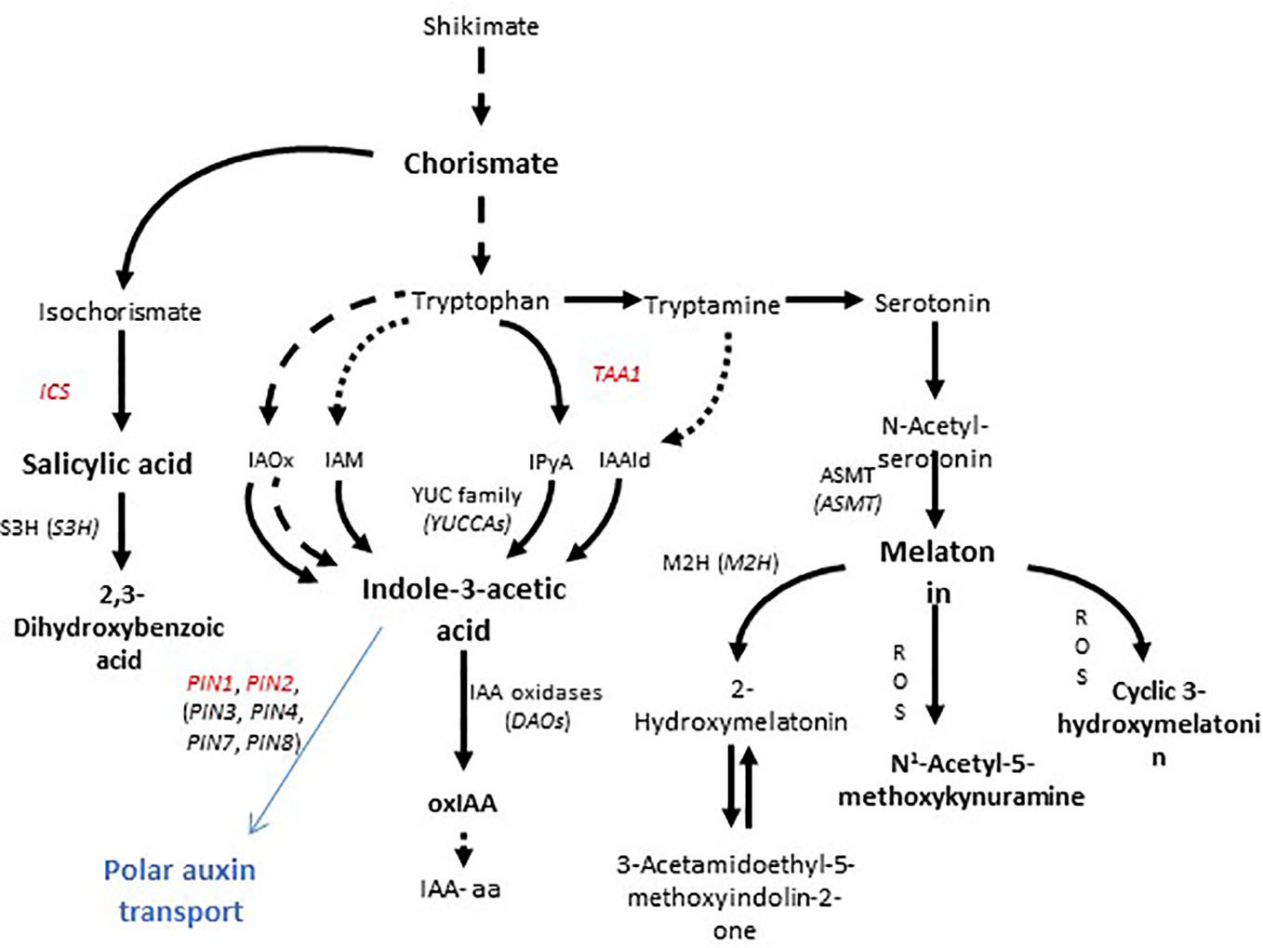
Biosynthesis of chorismate-derived phytohormones and efflux transport of auxin in plants. Salicylic acid is derived from isochorismate, while auxin and melatonin come from tryptophan, which is in turn synthesized from chorismate. Isochorismate synthase (ICS), tryptophan amino transferase (TAA1), and N-acetyl-serotonin methyl transferase (ASMT) play crucial roles in the biosynthesis of salicylic acid, indole-3-acetic acid (auxin), and melatonin, respectively. PIN proteins show asymmetrical localizations on the membrane and are responsible for polar auxin transport (shown in blue). Genes, the expression of which was measured in the present study, are indicated in red.

Auxins, salicylic acid, and melatonin are phytohormones involved in the signaling and regulation of many crucial processes in plants. Auxins have been widely described as growth and development regulators with multiple functions in plants, playing a key role in organ morphogenesis, apical dominance, adventitious rooting, and cell expansion, among other processes (see Taylor-Teeples et al., 2016). Salicylic acid is known to be involved in triggering the defense response against biotrophic and hemi-biotrophic pathogens (Loake and Grant, 2007) as well as having an important role in growth arrest under abiotic stress conditions (Carswell et al., 1989; Dong et al., 2014; Wani et al., 2017), although at low concentrations it can also promote lateral root formation (Pasternak et al., 2019). Finally, melatonin has not only been found to have auxin-like functions (Chen et al., 2009; Zuo et al., 2014; Wen et al., 2016) but it also has been suggested to act as a potential antioxidant in some specific plant organs and plant species (Arnao and Hernández-Ruiz, 2015) and a regulator of plant responses to pathogens (Chen et al., 2018). Interestingly, these three hormones share a common precursor—chorismate—thus a metabolic crosstalk occurs between them and a number of genes must be finely regulated to divert chorismate metabolism towards these compounds (**Figure 1**).

Indole-3-acetic acid (IAA), the major auxin bioactive form present in plants, can be produced from tryptophan or from tryptophan precursors in seemingly complex pathways that in all cases compete for the same chorismate precursor. Auxin promotes vegetative growth both above- and belowground by regulating cell expansion, cell differentiation, and organogenesis, while cell-cell polar transport mediated by PIN proteins regulated auxin distribution (Pacifici et al., 2015). Salicylic acid is a phenolic acid also derived from chrorismate (but not from tryptophan) that is not only involved in biotic stress response (mainly biotrophic pathogens) generally retarding plant growth and inducing pathogen-related genes (Qi et al., 2018), but also inducing adventitious root formation and improving root growth in particular under stress conditions (Armengot et al., 2014; Pasternak et al., 2019). Melatonin, another chorismate-derived compound, which competes for this common precursor with salicylic acid and auxin, and for tryptophan with auxin but not with salicylic acid, has also been shown to promote lateral root formation and development, by, among other mechanisms, modulating auxin response (Liang et al., 2017). In a previous study Pepton enhanced foliar levels of auxin, gibberellins and jasmonic acid in tomato leaves, whereas salicylic acid was not affected under conditions of water deficit stress (Casadesús et al., 2019).

The aim of this work was to assess to what extent an enzymatically hydrolyzed animal protein-based biostimulant (Pepton) can promote root growth and, if so, to also establish a mechanism of action, with an emphasis on the endogenous contents of chorismate-derived hormones. This is very important not only to promote growth and yield in several horticultural crops, but also to better understand the mechanism of action of enzymatically hydrolyzed animal protein-based biostimulants.

## Materials and Methods

### Growth Conditions, Treatments, and Samplings

Seeds of tomato (*Solanum lycopersicum*, var. “Ailsa Craig”), which were obtained from the Experimental Field Facilities of the University of Barcelona (Barcelona, NE Spain), were used for experiments. For experiment 1 (large roots), seeds were sown on May 23, 2019, in 1-L pots in a growth chamber (12 h light/12 h dark, at 21.9°C and 65% relative humidity). On June 11, 2019, seedlings were transferred to a closed hydroponic system prepared in an open-windows greenhouse situated at the Faculty of Biology of the University of Barcelona (Barcelona, NE Spain) with 12 plants for each container and 6 containers per treatment were established and filled with 21 L of half-strength Hoagland nutrient solution in (Hoagland and Arnon, 1938) distilled water (indicated on **Supplementary Table 1**). Treatments started after 2 weeks of growth in hydroponics, on June 25, 2019, when roots were sufficiently large to obtain high density of roots in the container to better produce nutrient deficiency by competition. Two treatments, control and Pepton, were imposed on plants at optimal temperature (24.6–25.51°C) in a background of nutrient deficiency (obtained by using half-strength Hoagland nutrient solution in a container with high plant density). Pepton was added and diluted on the nutrient solution at manufacturer’s recommendation dose (Pepton at 1.06 g/container, calculated using plant density on the area of the study as equivalent of 4 kg/ha) the first day of the experiment. For experiment 2 (small roots), seeds were sown on December 20, 2019, in 1-L pots in a growth chamber (12 h light/12 h dark, at 21.9°C and 65% relative humidity). On January 17, 2020, seedlings were transferred to a closed hydroponic system prepared in the same greenhouse and in the same way as described before situated at the Faculty of Biology of the University of Barcelona (Barcelona, NE Spain) with 12 plants for each container and three containers per treatment were established and filled with 21 L of full-strength Hoagland nutrient solution (indicated on **Supplementary Table 1**). Treatments started after three days of growth in hydroponics, on January 20, 2020, when roots were still small. We applied two treatments, control and Pepton, in a background of low-temperature stress (water temperature of 11.0°C to 12.6°C obtained by conditioning the greenhouse to decrease the indoor temperatures). In both treatments, we applied the full-strength nutrient solution but in the treatment of Pepton, we added and diluted this biostimulant on the nutrient solution following manufacturer’s recommendation dose (430 mg/container, calculated using plant density on the area of the study as equivalent of 4 kg/ha) the first day of the experiment. Samplings for both experiments were performed at days 1, 2, and 4 from the start of treatments at midday. At each time point, 1, 2, and 4 days from the start of treatments, the whole root system from 11 plants were collected in experiment 1 and from 5 plants in experiment 2, weighed and gently dried on a paper before being immediately frozen in liquid nitrogen and then kept at −80°C to be used for hormonal and gene expression analyses. **Table 1** contains the conductivity, pH, and temperature of the nutrient solution during experiments 1 and 2 for the different testing time (1, 2, and 4 days).

Table 1pH, conductivity and temperature of the nutrient solution during experiment 1 (A) and experiment 2 (B).

**Table:** **(A)** Experiment 1/nutrient deficiency (large roots).

Day	Treatment	Conductivity (µS/cm^2^)	pH	Temperature (°C)
1	Control	815	5.90	25.5
1	Pepton	978	6.55	25.5
2	Control	619	6.64	24.8
2	Pepton	1032	6.78	24.6
4	Control	653	7.23	25.2
4	Pepton	824	6.89	25.1

**Table:** **(B)** Experiment 2/suboptimal temperatures (small roots).

Day	Treatment	Conductivity (µS/cm^2^)	pH	Temperature (°C)
1	Control	2546	6.67	11.0
1	Pepton	2516	6.69	11.0
2	Control	2543	6.82	11.7
2	Pepton	2520	6.90	11.7
4	Control	2547	6.76	12.6
4	Pepton	2510	6.84	12.6

### Pepton Composition

Pepton is an animal protein enzymatic hydrolyzed product that contains high amounts of total organic matter (79%) and organic nitrogen (12%), with a carbon/nitrogen ratio of 3.6 (**Table 2**). The product is very rich in L-α amino acids (84.8%) and contains a high proportion of free amino acids (16.5%), iron (3000 mg/kg) and potassium (4.0%). The average molecular weight distribution of Pepton is around 2,000 to 3,000 Da, from which 66% of the peptides are considered short-chain amino acids (with less than 50 amino acids per chain) and 16% of peptides in Pepton are long-chain peptides (>50 amino acids). A complete chemical composition of this biostimulant can be found in Polo and Mata (2018). The manufacturing process of Pepton involved an enzymatic hydrolysis at high temperature (>50°C) at controlled conditions that hydrolyzed the animal protein. Following the hydrolysis process, the enzyme is inactivated by increasing the temperature and the final product is spray-dried at specific conditions that involved the treatment of >90°C throughout its substance. Final product is a hygienic, brown, granulated powder that meets all physical and microbiological requirements for being used as biostimulants in the agro industry.

**Table 2:** Composition of the enzymatically hydrolyzed animal protein-based biostimulant (Pepton).

	Pepton
Total organic matter, %	79.0
Total nitrogen, %	13.0
Organic nitrogen, %	12.0
Ammonia nitrogen, %	1.0
Carbon/Nitrogen	3.8
Potassium oxide (K_2_O), %	4.0
Phosphorous (P_2_O_5_), %	0.3
Calcium, mg/kg	300
Magnesium, mg/kg	500
Iron, mg/kg	3000
Amino acid composition	
Alanine, %	6.90
Arginine, %	3.22
Aspartic acid, %	9.93
Cysteine	<0.1
Glutamic acid, %	7.25
Glycine, %	4.06
Histidine, %	6.34
Isoleucine, %	0.15
Leucine, %	10.99
Lysine, %	7.19
Methionine; %	0.71
Phenylalanine, %	5.93
Proline, %	2.84
Serine, %	3.88
Threonine, %	2.47
Tryptophan, %	1.25
Tyrosine, %	1.92
Valine, %	6.79
	
Total amino acids, %	84.83
Free amino acids, %	16.52

### Chorismate-Derived Hormones

Chorismate-derived hormones, including salicylic acid, auxin, and melatonin were determined by liquid chromatography coupled to electrospray ionization tandem mass spectrometry (HPLC/ESI-MS/MS) as described by Müller and Munné-Bosch (2011). Salicylic acid and IAA (auxin) were analyzed using negative ion mode while melatonin was measured using positive ion mode. Extracts were performed using 100 mg of well powered fresh root of each plant with a mixture of methanol and acetic acid (99:1, v/v) as a solvent. Deuterium-labeled plant hormones were added to the extract being the 250-µL the final volume and the mixture was vortexed and ultra-sonicated for 30 min (Branson 2510 ultrasonic cleaner, Bransonic, Danbury, CT, USA). Then the extract was vortexed again and centrifuged at 4°C for 10 min at 1300 rpm. The supernatant was collected, filtered using 0.22-μm hydrophobic PTFE Syringe Filters (Phenomenex, Torrance, CA, US). Two extractions were done and the final extract was injected into the HPLC/ESI-MS/MS system.

### Estimation of Root Growth

Root growth of 11 plants for each treatment and time point was estimated by measuring the root weight of the whole root system on a dry matter basis for experiment 1 and those of five plants for the experiment 2. Dry weight was estimated after drying the tissue in the oven to constant weight at 80°C. In addition, root growth was also estimated by scanning the roots of five plants at the end of experiment 2 and using ImageJ software to calculate root area of each plant.

### Gene Expression Analyses

Three individuals of each treatment were randomly selected at each time point for gene expression analyses. Approximately, 100 mg of fresh tissue of each individual plant were ground in liquid nitrogen until obtaining a fine powder. Then, total RNA from roots was extracted using the Spectrum Plant Total RNA Kit (Merck) according to manufacturer’s instructions, including a DNase I digestion (On-Column DNase I Digestion Set, Merck). Yield and quality of an aliquot of denaturalized RNA for the qPCR analysis were tested with the Qubit fluorometer—using the Qubit™ RNA HS Assay Kit, Thermo Fisher Scientific—and the 2100 Bioanalyzer system (Agilent Technologies, California, USA), respectively. RNA was considered of high quality when RNA integrity number was equal or greater than five (Jeffries et al., 2014). Finally, RNA was reverse transcribed to cDNA with the RevertAid First Strand cDNA Synthesis Kit (Thermo Fisher Scientific) according to manufacturer’s instructions.

For specific primer design of *ICS2, TAA1*, and *PIN2* for *S. lycopersicum* var. Alisa Craig, coding sequences were acquired from the Sol Genomics Network (Ithaca, NY, USA) tomato database. Sequences were introduced in the FGENESH online program from Softberry portal (Mount Kisco, NY, USA), selecting the “generic *Tomato lycopersicum*” in the “organism” option. mRNA sequences were obtained and then introduced in Primer-BLAST from NCBI for primer design. Minimum size of the PCR product was set to 70 bp and maximum to 180 bp; minimum T_m_ was set to 60°C and maximum to 63°C; and, the “organism” was changed to “green plants.” Suggested sequences by NCBI of tomato were accepted, and then, the first pair of primers was selected after testing for formation of heterodimers with the OligoAnalyzer Tool from Integrated DNA Technologies (Coralville, IA, USA). Primers for the target gene *SlPIN1* and for the reference gene *EF1α* were directly selected from previous studies in tomato (Mascia et al., 2010; Gan et al., 2019, respectively). Primer sequences can be found in **Supplementary Table 2**.

Gene expression quantification was performed by RT-qPCR on cDNA using the Roche LightCycler^®^ 480 Instrument II (Roche, Basel, Switzerland) and LightCycler^®^ 480 SYBR Green I Master (Roche). The RT-qPCR reactions were set according to manufacturer’s instructions with some modifications. The final volume was adjusted to 10µl, and the reactions started with 5 min of incubation at 95°C followed by 45 cycles of 95°C for 10 s, 60°C for 20 s, and 72°C for 30 s. The RT-qPCR reactions ended with 5 s at 95°C and 1 min at 65°C. For each sample, RT-qPCR was performed with *SlICS2, SlTAA1, SlPIN1*, and *SlPIN2* as target genes primers and with *SlEF1α* as reference gene primers. Cp values and dissociation curves were analyzed after RT-qPCR reactions with the LightCycler^®^ 480 Software, Version 1.5 (Roche, Basel, Switzerland). All primers had an efficiency of *ca*. 2; therefore, relative expression levels were calculated with the 2^−ΔΔCT^ method and normalized with the Cp values of each gene at control conditions (control treatment and time 1 day after application).

### Statistical Analyses

To determine the effect of Pepton and time on root biomass, hormones and gene expression, a linear regression (*lm*) was used with “treatment/Pepton” and “time” as predictors. The *P* of the explanatory variables were estimated using conditional *F*-tests using the function Anova (*car* package). Differences were considered significant when *P* < 0.05. To meet normality and homoscedasticity of residues, which were visually checked as described by Zuur et al. (2009), data was either log or square root transformed before analyses. For Experiment 1 data, IAA and melatonin were square root transformed. For Experiment 2 data, root biomass was log transformed and salicylic acid and IAA were square root transformed. All statistical tests were performed using R statistical software (R Foundation for Statistical Computing, Vienna, Austria).

## Results

**Figure 2:**
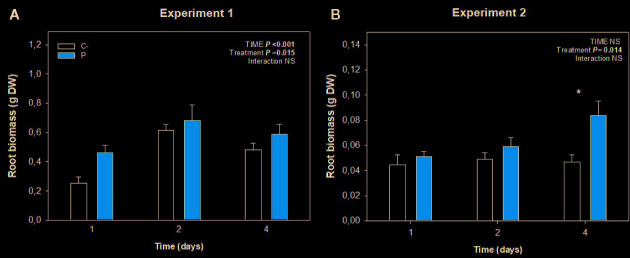
Variations in root biomass in Pepton-treated tomato plants compared to controls. Data are the means ± SE of **(A)** n=11 individuals for experiment 1 and **(B)** n= 5 for experiment 2. Significant effects of “treatment” and “time” were tested using conditional *F* tests (linear model with “treatment” and “time” as explanatory variables, *P* values below 0.05 are shown in bold in the inlets). Experiment 1 was conducted under nutrient deficiency and large root system and experiment 2 under sub-optimal temperature and small root system. *P < 0.05.

**Figure 3:**
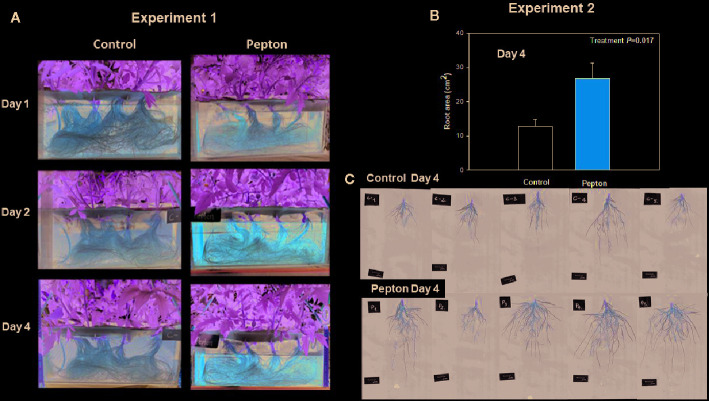
Changes in root morphology and area in Pepton-treated tomato plants compared to controls. **(A)** Images correspond to day 4 for experiment 1. **(B)** Root area measured at day 4 for experiment 2. Data are the means ± SE of 5 individuals. Differences between treatments on root area were tested with a one-way ANOVA (*P* values are shown in bold in the inlet). **(C)** Images correspond to day 4 for experiment 2. Experiment 1 was conducted under nutrient deficiency and large root system and experiment 2 under sub-optimal temperature and small root system.

Root growth was stimulated by the application of this biostimulant during both experiments (**Figure 2**), being more evident after 4 days in experiment 2 (low temperature conditions, **Figure 2B**). A more detailed analysis of root growth imaging revealed that Pepton might have promoted lateral root growth during experiment 1 under nutrient deficiency condition (with longitudinal growth being limited by the depth of the hydroponic tanks, **Figure 3A**). However, the biostimulant promoted both longitudinal and lateral growth in a longer term (4 days) in smaller plants during experiment 2 (without any limitation on longitudinal root growth since tanks of the same depth were used for both experiments). Therefore, the highest differences in root growth were observed at 4 days for experiment 2 with values 2-fold higher in Pepton-treated plants than in control plants (**Figures 2** and **3B, C**).

**Figure 4:**
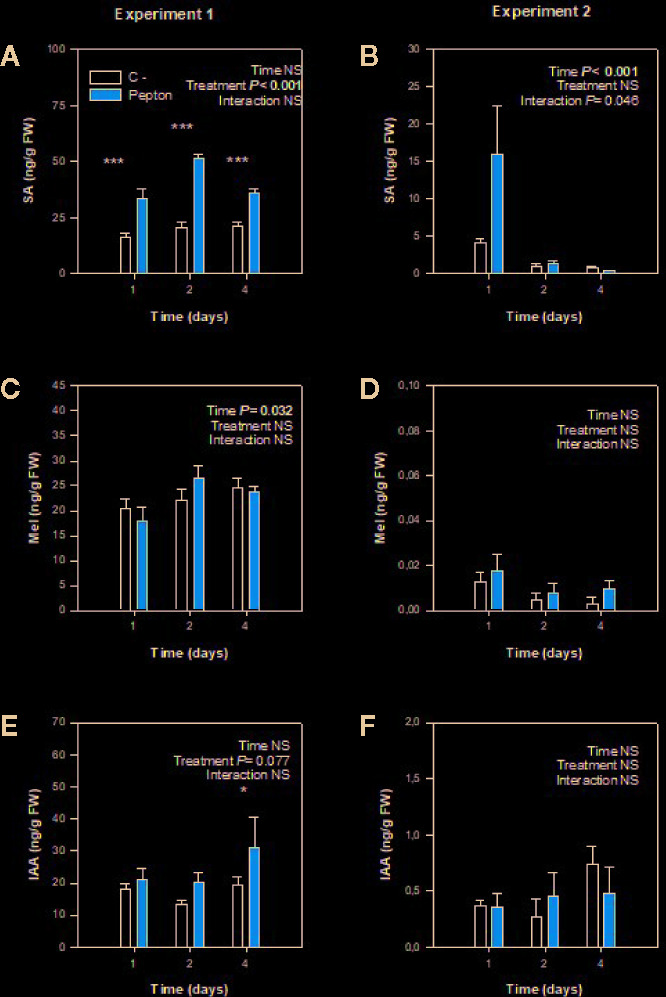
Variations in the endogenous concentration of chorismate-derived phytohormones in roots of Pepton-treated tomato plants compared to controls. **(A, B)** salicylic acid (SA), **(C, D)** melatonin (Mel), **(E, F)** indole-3-acetic acid (IAA). Data are the means ± SE of n = 5 and 11 individuals for experiments 1 and 2, respectively. Significant effects of “treatment” and “time” were tested using conditional *F*-tests (linear model with “treatment” and “time” as explanatory variables, *P* values below 0.05 are shown in bold in the inlets). Experiment 1 was conducted under nutrient deficiency and large root system and experiment 2 under sub-optimal temperature and small root system. *P < 0.01; ***P < 0.001.

The concentration of salicylic acid increased in Pepton-treated plants compared to control in all time points of experiment 1, using plants with large roots exposed to nutrient deficiency (*P* < 0.001, **Figure 4A**). In contrast, in experiment 2 (using plants with small roots exposed to suboptimal temperatures), the endogenous concentration of salicylic acid was similar between treatments, but on day 1 the salicylic acid concentration of Pepton-treated plants was higher than control plants (“Time x Treatment”, *P* = 0.046) (**Figure 4B**).

In both studies, melatonin concentration was not influenced by Pepton treatment (P > 0.10, **Figures 2C, D**). In case of auxin, in experiment 1 under nutrient deficiency conditions it seemed to occur a marginal increase in auxin concentration in Pepton-treated plants compared to the control (“Treatment” *P =* 0.077) (**Figure 4E**).

**Figure 5:**
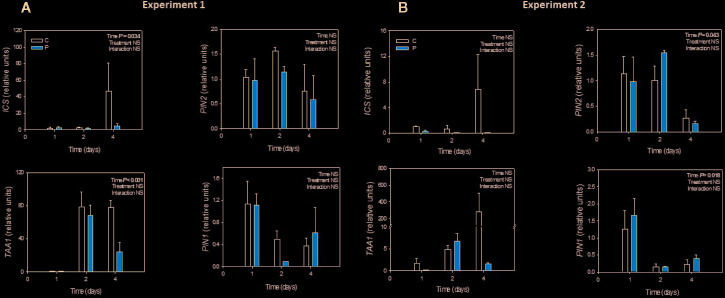
Variations in the relative expression of the genes encoding for isochorismate synthase (*ICS*), tryptophan amino transferase (*TAA1*) and PIN proteins (*PIN1* and *PIN2*) in Pepton-treated tomato plants compared to controls. **(A)** Experiment 1 and **(B)** Experiment 2. Data are the means ± SE of n = 3 individuals. Significant effects of “treatment” and “time” were tested using conditional *F* tests (linear model with “treatment” and “time” as explanatory variables). No significant or marginal differences in any of the tested genes were observed (*P* > 0.10). Experiment 1 was conducted under nutrient deficiency and large root system and experiment 2 under sub-optimal temperature and small root system.

Gene expression analyses revealed that neither *ICS*, *TAA1* nor *PIN* expression was influenced by Pepton application during the two experiments (**Figures 5A, B**).

## Discussion

Enzymatically hydrolyzed animal protein-based biostimulants, such as Pepton, may be an alternative to protein hydrolysates of plant origin since they can promote circular economy and bridge the current gap between recycling animal waste products and agriculture. Considering that in developing countries, between 50 to 60% by weight of farm animals are not profitable for human consumption, finding sustainable applications that improve the use of high-quality source of organic material will reverse in better use of the limited resources of nature. Amino acids and peptides derived from protein hydrolysates, either from plant or animal origin, may improve plant performance through various mechanisms, including effects on hormonal signaling (Du Jardin, 2015; Yakhin et al., 2017; Casadesús et al., 2019; Zulfiqar et al., 2019). In the present study, it was shown that not only an enzymatically hydrolyzed animal protein-based biostimulant (Pepton) can improve root growth in tomato plants, but that these effects may be mediated by chorismate-derived hormones, in particular by the involvement of salicylic acid or by sourcing aromatic amino acids as phenylalanine, the accumulation of which may also involve the production of salicylic acid.

### Pepton Might Improve Lateral Root Growth Through Modulation of Salicylic Acid Contents

Recent studies suggest that biostimulants based on protein hydrolysates improve crop performance by stimulating carbon, nitrogen, and hormonal metabolism of plants including tomato (Colla et al., 2014; Colla et al., 2017; Casadesús et al., 2019). In the present study, Pepton treatment led to a significant effect on the endogenous concentrations of the chorismate-derived phytohormone salicylic acid.

Salicylic acid is a stress-related phytohormone, playing a crucial role in biotic stress responses, being the major hormone involved in systemic acquired resistance (Fu and Dong, 2013). Furthermore, salicylic acid is a typical growth inhibitor, as it occurs with other stress-related phytohormones, due to the commonly accepted trade-off between defense and growth (Van Butselaar and van den Ackerveken, 2020). However, phytohormones are involved in a large number of developmental processes and salicylic acid is not an exception. Indeed, salicylic acid is involved in signaling of lateral root initiation and growth interacting with auxin in a dose-dependent manner (Pasternak et al., 2019). Therefore, under nutrient deficiency, increased salicylic acid contents may represent a basic mechanism to explore new soil environments and face poor soils, in our case, probably fighting against the limits of the hydroponics container, so that this phytohormone can have an impact on crop performance and yield (Nibau et al., 2008).

In previous studies, it has been observed an enhanced growth due to an auxin-like activity for protein hydrolysates products of plant origin (Colla et al., 2014; Ugolini et al., 2015; Elzaawely et al., 2016; Ertani et al., 2017), but Pepton did not contain nor seemed to largely influence auxin concentrations, thus other mechanisms might explain these positive effects on root growth.

On the one hand, the effects of Pepton might be related to a direct influence of amino acids, which can improve nitrogen metabolism and promote root growth. In fact, the abundance of nitrogen compounds can have a huge impact on root architecture and on lateral root formation (Nibau et al., 2008). On the other hand, Pepton effects might be related to an enhanced accumulation of salicylic acid concentrations, which might induce lateral root formation, in particular during experiment 1 (Shi et al., 2010; Armengot et al., 2014; Pasternak et al., 2019), in order to explore new soil and solve suboptimal nutrient environment situation. But, since melatonin and auxin concentrations did not increase in response to Pepton application in experiment 2, to understand the longitudinal root growth promotion observed, we evaluated the underlying mechanism through an examination of possible changes in polar auxin transport, an aspect that was investigated at the gene expression level.

Using protein hydrolysates of plant origin, Ertani et al. (2017) demonstrated higher expression of several ethylene/jasmonates/abscisic acid responsive genes including wound-induced proteins and heat-shock proteins which are crucial in both biotic and abiotic stress response; however, to our knowledge no studies have investigated changes in gene expression upon the application of an enzymatically hydrolyzed animal protein-based biostimulant. We expected that the observed significant changes in salicylic acid concentration affected gene expression of at least some of the genes related with the synthesis of salicylic acid (*ICS*), or the synthesis of auxin (*TAA1*) or the auxin transport (*PINS*) in accordance with the mechanism of salicylic acid involvement in lateral root growth described by Pasternak et al. (2019). However, gene expression analyses revealed that neither *ICS*, *TAA1* nor *PIN* expression was influenced by Pepton application during the two experiments (**Figures 5A, B**). Despite we did not find statistical differences, probably because of high interindividual variability, data suggest a tendency of Pepton to downregulate *ICS* and *TAA1* expression at day 4 in both experiments. It has been shown a negative regulation of *ICS* expression by SA in *Arabidopsis thaliana* exposed to ozone (Ogawa et al., 2007) and auxin was also proposed to play a role in negative feedback regulation of *TAA1* in Arabidopsis (Suzuki et al., 2015), facts that could be a possible explanation for the results in experiment 1.

### A Model Linking an Enzymatically Hydrolyzed Animal Protein-Based Biostimulant (Pepton) With Improved Root Growth

**Figure 6:**
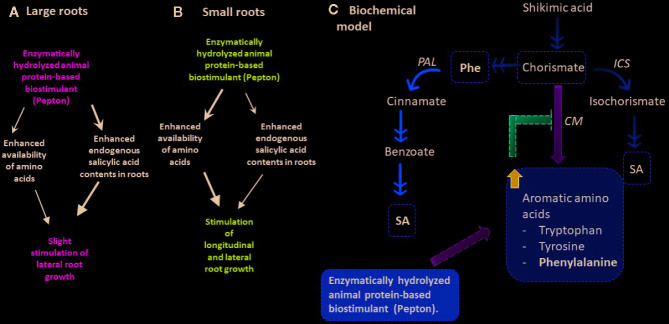
Model proposed to explain the molecular mechanism of action of an enzymatically hydrolyzed animal protein-based biostimulant (Pepton). **(A)** Experiment 1, plants with large roots exposed to nutrient deficiency, **(B)** Experiment 2, plants with small roots exposed to suboptimal temperatures. Note that the hormonal pathway (salicylic acid increases) appeared to play a major role in experiment 1 than in experiment 2. **(C)** Integrated model proposed to explain the increased salicylic acid content. Note that an increased Phe availability provided by Pepton may stimulate diversion of the salicylic acid pathway through PAL instead of ICS. Orange arrows represent biosynthetic pathway steps, green and red arrows represent positive or negative relationships, respectively. Blue arrow represents an increase on the content of aromatic amino acids. Phe, phenylalanine; PAL, phenylalanine ammonia lyase; ICS, isochorismate synthase; CM, chorismate mutase.

Overall, mechanisms of action of biostimulants are based on an enhancement of key physiological responses of plants to improve their development and yield. Regulation of gene expression, stimulation of amino acid biosynthesis, and increases of antioxidants, osmolytes, protein, or pigment contents have been hypothesized as mechanisms of action. In general, metabolic and hormonal effects, an improvement of nutrition efficiency, and physiological response to abiotic stress and to biotic stress have been reported (Yakhin et al., 2017). Casadesús et al. (2019) demonstrated positive effects of enzymatically hydrolyzed animal protein-based biostimulant (Pepton) on antioxidant system (plastochromanol-8), growth-promoting phytohormones auxins, cytokinins, and gibberellins, and defense-related phytohormones (jasmonic acid) in leaves of tomato plants under water stress, suggesting that this product acts by reducing the negative impact of stress and that the amino acid composition of the product (Phe, Tryp, and Tyr) may lead to the increases of both antioxidants and phytohormone observed since there are metabolic connections between Phe and Trp and IAA or between Tyr and tocochromanols. Our results are in agreement with this idea since under suboptimal conditions, reduced nutrient availability or low temperatures in experiments 1 and 2, respectively, Pepton exerted a positive effect on root growth, reducing the negative impact of stress. While in the leaves of plants under water stress Pepton affected auxin but not SA, in the roots of plants under nutrient deficiency Pepton affected mainly SA contents. This can be understood considering that plant responses to stresses are stress and organ specific so in each situation the plant stress response and the contribution of Pepton in alleviating negative impact of stress were differentially coupled. In the present study, it seemed that different mechanisms could be involved in the observed stimulation of root growth, both primary and lateral, by the biostimulant (**Figure 6**). First, as the product is a hydrolyzed protein-based product, it can be a source of amino acids, increasing their availability to the plant and this results in the stimulation of both primary and lateral growth observed in experiment 2 (in which roots had no restriction in longitudinal root growth, **Figure 6A**). These observed changes in root growth might be related to the effects of the biostimulant promoting nitrogen metabolism with possible changes in expression of nitrogen-related genes, as Santi et al. (2017) had reported using protein hydrolysates in maize seedlings. They reported that the presence of peptides rather than free amino acids was important to the effect on root growth highlighting a specific role of small peptides (1500-2000 Da) on the regulation of root growth. Peptides of the enzymatically hydrolyzed animal protein-based biostimulant (Pepton) are around 2000-3000 Da, from which 66% of them are considered short-chain peptides, thus they might exert a positive effect on regulation of root growth. However, this remains to be proved as we did not analyze the nitrogen metabolism in this study.

Second, when longitudinal root growth was physically restricted in experiment 1 and plants were exposed to nutrient deficiency, amino acids appeared not only to be used for nitrogen metabolism as proposed above but also to produce enhanced endogenous concentrations of salicylic acid (“Treatment” *P* < 0.001), with a marginal effect on auxin concentrations (“Treatment” *P =*0.077) (**Figures 2A, E**) to induce lateral root growth as a response to the stressful conditions (**Figure 6B**). This enzymatically hydrolyzed animal protein-based biostimulant contains aromatic amino acids, such as tryptophan, tyrosine, and phenylalanine (which are derived from chorismate). The possible increased availability of amino acids might lead to chorismate accumulation since the activity of chorismate mutase (which is the first enzyme in the production of phenylalanine from chorismate) is known to be sensitive to feedback inhibition by phenylalanine accumulation (Gilchrist and Kosuge, 1980). That supposed accumulation of chorismate and increased availability of phenylalanine, the substrate of phenylalanine ammonia lyase (PAL), could induce the production of salicylic acid by the PAL biosynthetic pathway (**Figure 6C**; Dempsey et al., 2011). This might particularly occur in experiment 1, with restriction in longitudinal root growth, thus in this regard, salicylic acid demand could be increased due to the stress conditions caused by nutrient starvation in addition to the longitudinal limitation in root growth. Therefore, the stimulation of root growth shown here by an enzymatically hydrolyzed animal protein-based biostimulant (Pepton) could be explained by the supposed enhanced availability of amino acids used by the plant in two main different ways. In one way, it seemed that plants could use these extra amino acid availability directly for growth (in addition with possible small peptide regulation on root growth) when no growth restriction was present (experiment 2) and, additionally, to produce salicylic acid (probably through the PAL biosynthetic pathway) when stress conditions and restriction of longitudinal growth were present (experiment 1, **Figure 6**). The marginal increases in auxin observed during experiment 1 (“Treatment” *P* = 0.077) might also be facilitated by an enhanced availability of the auxin precursor, tryptophan (**Figure 1**).

## Conclusion

It is concluded that an enzymatically hydrolyzed animal protein-based biostimulant (Pepton) exerts a positive effect on the root growth of tomato plants by stimulating salicylic acid accumulation and enhancing lateral root growth. In addition, we propose that the enhanced salicylic acid content might be explained by the presence of aromatic amino acids, in particular of Phe, in Pepton composition, which can divert salicylic acid production through PAL independent of chorismate. Further research is needed to explore the functional contribution of changed hormone levels versus nutritive effects of Pepton and other enzymatically hydrolyzed animal protein-based biostimulants on root development and growth.

## Data Availability Statement

The raw data supporting the conclusions of this article will be made available by the authors, without undue reservation, to any qualified researcher.

## Author Contributions

JP and SM-B conceived and designed the experiments with the help of AC. MP-L performed and wrote the statistical analyses. AC, and MP-L performed experiments. AC prepared figures. SM-B wrote the first draft of the manuscript with the help of AC, MP-L, and JP. JP and AC prepared the final manuscript with the help of SM-B. All authors contributed to the article and approved the submitted version.

## Funding

This study was partially funded by the European Union Regional Development Fund within the framework of the ERDF Operational Program of Catalonia 2014-2020 with the reference RD17-1-0096 (Generalitat de Catalunya, Spain) and by APC Europe, S.L. The funders were not involved in the study design, collection, analysis, interpretation of data, the writing of this article or the decision to submit it for publication.

## Conflict of Interest

JP is employed by APC Europe, S.L., Granollers, Spain. This does not alter our adherence to all the Frontiers Plant Science policies on sharing data and materials. APC Europe, S.L. manufacture and sells Pepton 85/16, the biostimulant used in this experiment.

The remaining authors declare that the research was conducted in the absence of any commercial or financial relationships that could be construed as a potential conflict of interest.
