# Supplementary material for: An Enzymatically Hydrolyzed Animal Protein-Based Biostimulant (Pepton) Increases Salicylic Acid and Promotes Growth of Tomato Roots Under Temperature and Nutrient Stress
Source: Front Plant Sci. 2020 Jul 1;11:953. doi: 10.3389/fpls.2020.00953 (PMC7342040; doi:10.3389/fpls.2020.00953)
Supplement: Supplementary file 1 [file Table_1.docx]

**Supplementary Table 1**. Nutrient composition of half- and full-strength Hoagland’s solution. Prepared as described Hoagland and Arnon (1938).

| **mM** | **NO_3_^-^** | **NH_4_^+^** | **PO4^-X^** | **K^+^** | **Ca^++^** | **Mg^++^** | **SO_4_^-2^** |
| --- | --- | --- | --- | --- | --- | --- | --- |
| half- strength | 7 | 1 | 1 | 3 | 2 | 0.5 | 0.5 |
| full-strength | 14 | 2 | 2 | 6 | 4 | 1 | 1 |
| **μM** | **Fe** | **Mn** | **Zn** | **B** | **Cu** | **Mo** |  |
| half- strength | 10 | 1 | 1 | 12.5 | 0.25 | 0.25 |  |
| full-strength | 20 | 2 | 2 | 25 | 0.5 | 0.5 |  |
