# Supplementary figures and images for: An Enzymatically Hydrolyzed Animal Protein-Based Biostimulant (Pepton) Increases Salicylic Acid and Promotes Growth of Tomato Roots Under Temperature and Nutrient Stress
Source: Front Plant Sci. 2020 Jul 1;11:953. doi: 10.3389/fpls.2020.00953 (PMC7342040; doi:10.3389/fpls.2020.00953)

**Supplementary Table 2**. Primers sequences for the RT-qPCR

**
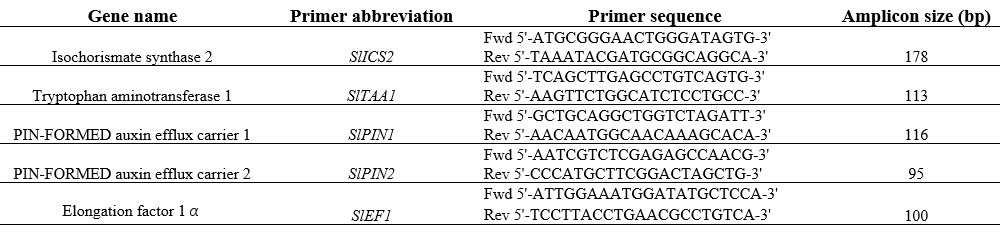
**

Supplement: Supplementary file 2 [file Table_2.docx]
